# Infection of Female Primary Lower Genital Tract Epithelial Cells after Natural Pseudotyping of HIV-1: Possible Implications for Sexual Transmission of HIV-1

**DOI:** 10.1371/journal.pone.0101367

**Published:** 2014-07-10

**Authors:** Yuyang Tang, Alvin George, Franklin Nouvet, Stephanie Sweet, Nkiruka Emeagwali, Harry E. Taylor, Glenn Simmons, James E. K. Hildreth

**Affiliations:** 1 Department of Molecular and Cellular Biology, College of Biological Sciences, University of California Davis, Davis, California, United States of America; 2 Department of Obstetrics and Gynecology, University of California Davis, Davis, California, United States of America; 3 Department of Microbiology and Immunology, Center for AIDS Health Disparities Research, Meharry Medical College, Nashville, Tennessee, United States of America; Institute of Infection and Global Health, United Kingdom

## Abstract

The global AIDS pandemic continues to expand and in some regions of the world, such as southern Africa, the prevalence of HIV-1 infection exceeds 20%. The devastating spread of the virus in young women in these countries appears disproportional to overall risk of infection. Regions with high prevalence of HIV-1 are often also highly endemic for other pathogenic viruses including HSV, CMV and HTLV. We propose that acquisition by HIV-1 of the envelope glycoproteins of other viruses, in a process we call “natural pseudotyping,” expands the cellular tropism of HIV-1, enabling it to infect female genital epithelial cells directly and thereby dramatically increasing risk of infection during sexual intercourse. In this proof-of-concept study, we demonstrate that when HIV-1 co-infects T cells along with the gammaretrovirus xenotropic murine leukemia virus-related virus (XMRV), progeny HIV-1 particles are produced capable of infecting primary vaginal, ectocervical and endocervical epithelial cells. These cell types are normally resistant to HIV-1 infection. Infection of primary genital cells was neutralized by antisera against the XMRV glycoprotein, confirming that infection was mediated by the XMRV glycoprotein acquired through pseudotyping of HIV. Inhibition by AZT showed that active replication of HIV-1 occurred in these cells and ruled out non-specific endocytic uptake of the virus. These results demonstrate that natural pseudotyping can expand the tropism of HIV-1 to include genital epithelial cells and have potential implications for sexual transmission of the virus.

## Introduction

The HIV/AIDS pandemic is primarily sustained by heterosexual transmission of HIV-1 and more than half of all new infections occur in young women. The prevalence of HIV-1 in some regions of Africa has exceeded 20% [1] and in sub-Saharan Africa, females constitute 75% of infected individuals between the ages of 15 and 24. In the nine countries in southern Africa most affected by HIV-1, prevalence among these young women was on average about three times higher than among men of the same age [2]. The virus is spreading among women at a rate that cannot be explained by other non-viral sexually transmitted diseases (STDs), sexual practices, including frequency and type of sex, or unusual virus characteristics [1]–[3]. While the overall risk of HIV transmission during heterosexual intercourse has been estimated to be 1 in 1000 to 1 in 200 [4], [5], anecdotal and clinical reports exist of “super spreaders” of HIV-1 who appear able to transmit HIV-1 to their sex partners quite efficiently [6]–[8].

The cellular tropism of retroviruses is determined by the cell receptor specificity of their envelope glycoproteins. In the case of HIV-1, the attachment protein gp120 binds specifically and sequentially to CD4 and chemokine receptors, primarily CXCR4 and CCR5 and this limits the tropism of HIV-1 to CD4^+^ T cells, macrophages, and dendritic cells [9]–[17]. Retroviruses such as HIV-1 are capable of incorporating envelope glycoproteins of other viruses. In a process known as pseudotyping, the envelope gene (gp160) of HIV-1 is deleted from the viral genome which is then expressed in cells expressing the envelope glycoprotein from other viruses. Importantly, the cellular tropism of the pseudotyped virus is that of the virus from which the envelope glycoprotein is derived. Thus, VSV G-pseudotyped HIV-1, like VSV, can infect many cell types, including epithelial cells. *In vivo*, it is well known that individuals infected by HIV-1 often harbor infection by a number of other viruses [18]–[23]. Co-infection of HIV-1 permissive cells (CD4+ T cells, macrophages, and dendritic cells) by HIV-1 and other viruses may allow HIV-1 to acquire the envelope glycoprotein (and cellular tropism) of the co-infecting virus. We refer to this phenomenon as “natural pseudotyping” to distinguish it from pseudotyping through molecular genetic techniques as described above. We propose that this process may represent a mechanism that profoundly increases the likelihood of sexual transmission of HIV-1.

The mucosal surfaces of lower regions of the female reproductive tract, comprised of squamous or columnar epithelial cells, represent a mechanical barrier to HIV-1. These cells do not express CD4 or CCR5 and are resistant to HIV-1 infection. How HIV-1 interacts with and crosses the genital mucosal epithelium to infect immune cells is incompletely understood. If HIV-1 acquires glycoproteins from epithelial cell-tropic viruses by co-infection with such viruses and these pseudotyped viruses are released into seminal fluid, direct infection of genital epithelial cells by HIV-1 could occur. Once HIV-1 has infected epithelial cells, spread to intraepithelial T cells, macrophages or dendritic cells followed by trafficking of these cells to lymph nodes would result in systemic infection. Therefore, we propose that natural pseudotyping may represent a mechanism that facilitates sexual transmission of HIV-1.

Here we report evidence to support the natural pseudotyping hypothesis in a model system employing xenotropic murine leukemia virus-related virus (XMRV), as the co-infecting virus. XMRV is a gammaretrovirus that was originally believed to be associated with prostate cancer and chronic fatigue syndrome, but recent reports indicate it arose as a result of a recombination event between two endogenous murine leukemia viruses after xenotransplantation of cells in nude mice [24], [25]. We chose XMRV as a model for natural pseudotyping with HIV-1 because of its broad cellular tropism, which overlaps with that of HIV-1 (T cells and macrophages). Furthermore, we demonstrated that lipid rafts play a role in the biology of this virus [26]. This observation further suggested XMRV as a good model virus for studying natural pseudotyping of HIV-1 given that HIV-1 assembly and release also occurs at lipid rafts [27]–[34], We explored whether XMRV and HIV-1 co-infection in T cells results in pseudotyped HIV-1 capable of infecting epithelial cell lines and primary cells from female lower genital tract epithelium. The results confirm that natural pseudotyping of HIV-1 occurs and expands its tropism to include genital epithelial cells.

## Materials and Methods

### Cell Lines

The LNcap cell line was purchased from American Type Culture Collection (Rockville, MD). LNcap cells and CEMX174 cells were maintained in cRPMI [RPMI 1640 (Gibco-BRL/Life Technologies, Gaithersburg, MD) supplemented with L-glutamine, 10 mM HEPES (pH 7.2), and 10% fetal bovine serum (FBS) (Hyclone, Logan, UT)]. CD4^+^ T cells were isolated from PBMC by negative selection with magnetic beads (Miltenyi Biotec) and were cultured in RPMI supplemented with 10% FCS (HyClone), 100 µg/mL streptomycin, and 100 U/mL penicillin. CD4^+^ T cells were activated with phytohemagglutinin (PHA) (3 ug/ml) in the presence of interleukin-2 (IL-2) (20 U/mL; Roche Applied Science) for 72 hrs. Human vaginal epithelial cell line VK2 (ATCC2616), ectocervical epithelial cell line Ect1/E6E7 (ATCCCRL1614) and endocervical cell line End1/E6E7 (ATCCCRL2615) were obtained from the American Type Culture Collection. These cells were selected because they were generated from normal vaginal, ectocervical and endocervical mucosal tissues; VK-2 cells and Ect1/E6E7 cells are derived from squamous epithelial cells lining the vagina and ectocervix respectively. End1/E6E7 cells are derived from simple columnar endocervical epithelium cells [35]. VK2, Ect1/E6E7 and End1/E6E7 cells were maintained in keratinocyte serum-free complete media (KSFM) (Gibco, Grand Island, NY).

### Primary Epithelial cells

Vaginal and cervical tissues from pre-menopausal women aged 18–44 years undergoing hysterectomy and/or vaginal vault repair were processed within 2–4 h of completion of surgery. The experimental protocol including collection of discarded tissue had full University of California, Davis, Institutional Review Board (IRB) approval. Signed informed consent forms were obtained from donors for all tissues used in this study. Tissues were collected in Human Vaginal Epithelial Cell Culture Complete Growth Media containing 0.25 µg/ml fungizone (Celprogen, San Pedro, CA). The underlying connective stromal tissue was carefully dissected away from epithelial layers. The tissue was cut into 1×1-cm pieces and placed with the epithelium surface facing down in 12-well culture plates to allow epithelial cell migration from the tissues. EpiGRO Human Epidermal Keratinocyte Complete Media (Millipore, CA) was added to each well. The medium was changed every other day until an epithelial monolayer was formed (3 to 7 days); the cells were then subjected to subculture and were expanded for cryopreservation and for use in HIV-1 infection studies. Cultures of human primary vaginal epithelial cells (Celprogen, CA) and human primary cervical epithelial cells (Cell Application, CA) were also purchased and maintained in medium provided by the cell vendor following vendor protocols.

### HIV-1 and XMRV infection of T cells

HIV-1 IIIB, HIV-1Bal and XMRV virus stocks were prepared from Jurkat cells, PM1 cells and LNcap cells, respectively. Culture supernatants from infected cells were collected, centrifuged at 1,000×*g* to remove cell debris, filtered through a 0.45-µm filter, and concentrated by ultracentrifugation at 100,000×*g* for 1 hr. The pelleted virus was resuspended in RPMI1640 containing 10% FBS, aliquoted, and stored at −80°C. The viral titers were measured by anti-p24 Gag ELISA (HIV) or by RT activity assay (HIV, XMRV) with a commercial kit (Cavidi Tech AB, Uppsala, Sweden). CEMX174 or primary CD4+ T cells were infected with XMRV and HIV-1 simultaneously or infected with XMRV first, followed by superinfection with HIV-1 by spinoculation as described previously (36). Cells infected with either virus alone were included as controls. Twenty-four hrs after exposure to HIV-1, the input virus was removed by thorough washing. The virus-containing supernatants were collected every two days for the next 4–8 days. The supernatants were centrifuged at 1,000×*g* to remove cells and cell debris, filtered through a 0.45-µm filter and then the viruses were pelleted by ultracentrifugation at 100,000×*g* through a 20% sucrose cushion and stored at −80°C. The viral titers were measured by anti-p24 Gag ELISA (HIV-1) or by quantitative RT PCR (qRT-PCR) (HIV-1, XMRV). Western blot analysis was performed to measure both XMRV and HIV-1 viral release into supernatants, as described previously [37].

### Viral infection of epithelial cells and immunofluorescence staining

Epithelial cells were grown on coverslips or 35-mm glass-bottom dishes to a density of 1 to 5×10^4^ cells/35 mm dish. Cells were exposed to the virus-containing supernatants or purified virus from HIV-1-infected, XMRV-infected, or HIV-1/XMRV co-infected T cells for 24 hrs; the input virus was then removed by thorough washing. For cell-associated infection, infected CEMX174 cells and the mock infected CEMx174 control cells were treated with 100 µg/ml of mitomycin C at 37°C for 1 hr and washed. The CEMx174 cells and primary epithelial cells were then co-cultured for 2 days at a ratio of 1∶1. The non-adherent CEMx174 cells were removed by washing the epithelial monolayers three times with PBS and growth media was then added back to the epithelial cells. For both cell free and cell-associated infection, the epithelial cell supernatants were harvested 5 days post-infection. The adherent epithelial cells were then fixed with 2% paraformaldehyde for 30 minutes and suspended in blocking buffer (5% normal goat serum and/or 1% mouse serum, 3% BSA in PBS), before permeabilization with 0.2% saponin. Primary antibody against HIV-1 Gag p24 (KC57-FITC, Beckman Coulter, Inc., Fullerton, CA) was incubated with the cells for 1 hr and the cells were then washed. For double-staining in primary epithelial cells, the cells were first incubated with anti-Cytokeratin 19 primary antibody (Sigma, CA). Secondary antibody binding and amplification of signal was accomplished with Cy3 goat anti-mouse IgG. Following incubation with secondary antibody, the cell monolayer was then incubated with FITC-conjugated anti-HIV Gag antibody as described above. Additional antibodies were also used in the characterization of primary epithelial cells. These included: anti-Cytokeratin 18, anti-Cytokeratin 8 (Sigma, CA); anti-XPR1 (Abcam, CA); anti-CD45, anti-CCR5, anti-CXCR4, and anti-CD4 (BD Biosciences, CA). After staining, the cells were examined with a Nikon TE2000 C1 laser scanning confocal microscope (Nikon Instruments, NY) or Olympus FV1000 laser scanning confocal microscope (Olympus, USA).

### Quantitative analysis of viral RNA

XMRV and HIV-1 viral RNA in supernatants from infected cells was isolated with QIAamp Viral RNA kits (Qiagen, CA) and subjected to reverse transcription with SuperScript III cDNA synthesis kits (Invitrogen, Grand Island, NY, STATE). Real-time PCR was performed using iQSYBR green supermix (Bio-Rad, Hercules, CA) (XMRV primers: 5′-AACCGTATGGCAGATCAAGC-3′ and 5′-TTTGCCTTGTAGGACCCAAT-3′) (HIV-1 Gag Primers: 5′-GGA GCT AGA ACG ATT CGC AGTTA-3′ AND 5′-GGT TGT AGC TGT CCC AGT ATT TGTC-3′). Standard curves were generated using viral stocks with known genome copy numbers. Real-time PCR assays were performed in an iCycler (BioRad) with iQ Sybr green supermix (Bio-Rad). All analyses were performed at least three times, with triplicate samples in each experiment.

### Zidovudine (AZT) treatment and antibody neutralization assay

Epithelial cells were plated and cultured overnight as described above. HIV-1-infected, XMRV-infected or HIV-1/XMRV co-infected CEMX174 cells or free virus from these infected cells were pre-incubated for 1 hr at 37°C with 10 µM of AZT or with dilutions of polyclonal goat anti-Friend MLV antibody (ATCC catalog #VR1537AS-Gt). Normal Goat serum and HIV-1 gp41 monoclonal antibody 2F5 (The NIH AIDS Reagent Program) served as controls. The viral mixtures were then added to epithelial cells and 24 hr later input virus or non-adherent cells were removed by washing 3 times with PBS. After adding back growth medium, the epithelial cells were incubated for another four days and immunofluorescence staining was performed to detect HIV-1 Gag expression. Culture supernatants were collected to measure XMVR and HIV-1 RNA by q-PCR or HIV-1 by Gag ELISA (HIV).

### Data analysis

All experiments were repeated a minimum of three times. Comparisons between two groups were performed by Student’s *t* test.

### Ethics Statement

None of the viruses used in these studies were collected directly from infected patients. They were obtained from collections maintained by the National Institutes of Health AIDS Reagent Program.

## Results

### HIV-1 produced in XMRV and HIV-1 co-infected CD4+ T cells infects epithelial cells

**Figure 1:**
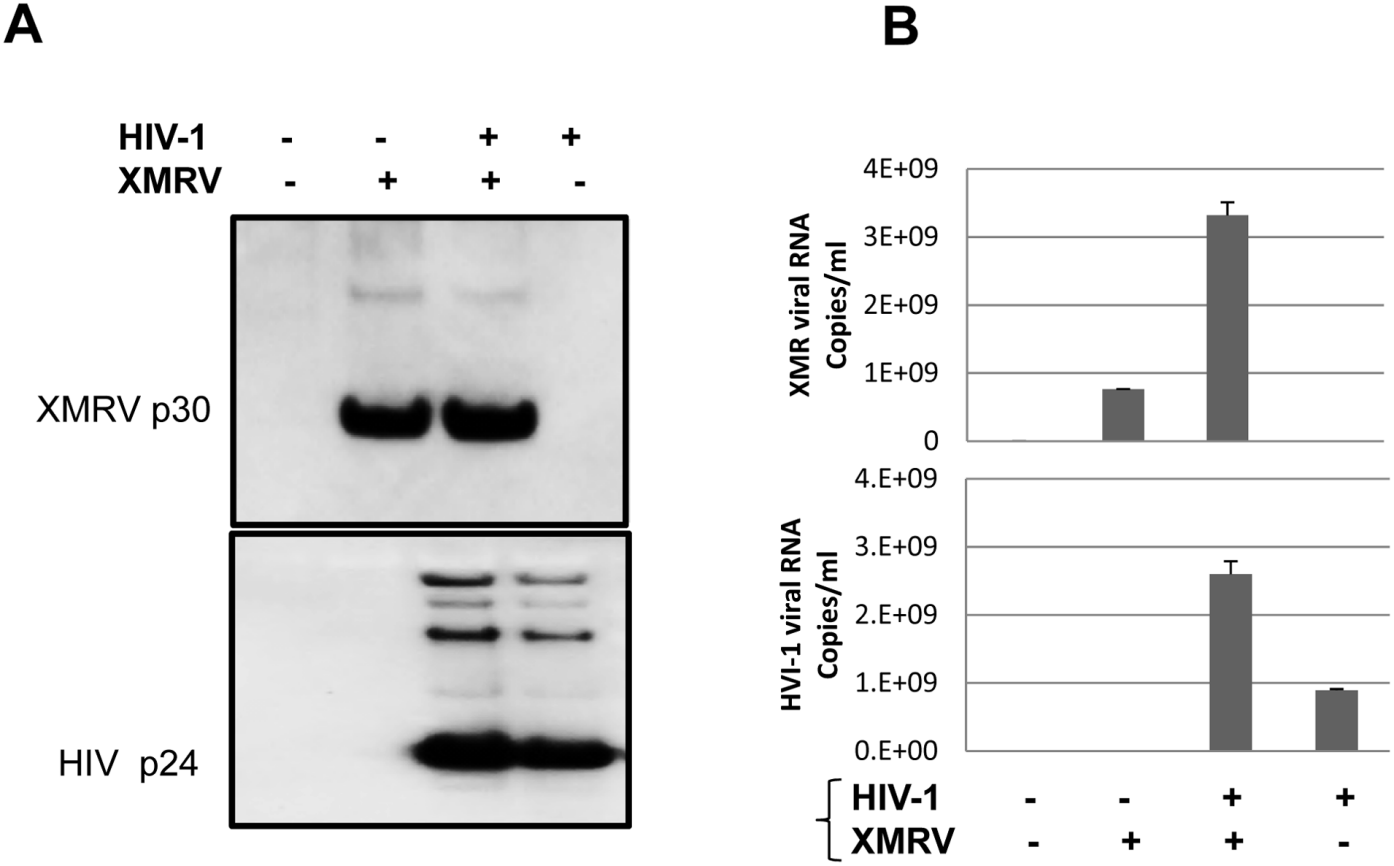
CEMx174 cells support both XMRV and HIV-1 replication. **A.** Supernatants from CEMx174 cells infected with the viruses indicated were collected 3 days post-infection and subjected to Western blot analysis with goat anti-serum against MLV Gag (top panel) or with an anti-HIV-1 Gag mAb (bottom panel). Data are representative of 5 independent experiments. **B.** Supernatants from CEMx174 cells infected with the viruses indicated were subjected qPCR to quantify XMRV and HIV-1 RNA genomes. Values represent an average of 5 experiments.

We sought to determine if natural pseudotyping of HIV-1 occurs when it co-infected the same cells along with the gammaretrovirus XMRV. In previous studies we demonstrated the involvement of cholesterol and lipid rafts in the biology of XMRV [26] as previously shown for HIV-1. XMRV utilizes the XPR1 membrane protein as its receptor; because this protein is widely expressed on cells of multiple lineages, the cellular tropism of the virus is broad and includes epithelial cells. The tropism of XMRV overlaps with that of HIV-1 and XMRV infection of human primary T cells has been demonstrated [38]. XMRV was thought to be associated with human disease at the time we began our studies but was subsequently shown to be a laboratory artifact [25]. To demonstrate that HIV-1 and XMRV can efficiently replicate and be released from co-infected cells, we infected CEMX174 cells and primary CD4+ T cells with XMRV, then superinfected with HIV-1 IIIB. In some experiments the cells were infected with both viruses simultaneously. Cells infected with either virus alone and uninfected cells served as controls. Twenty-four hrs after adding HIV-1, the input virus was removed by extensive washing and HIV-1 and XMRV production was assessed after 3 additional days of culture. As shown in Figure 1A, western blot analysis confirmed that HIV-1 was released from HIV-1-infected and XMRV/HIV-1 co-infected cells. Similarly, XMRV p30 was detected in the supernatants from XMRV-infected and HIV-1/XMRV co-infected cells.

We also performed real-time PCR analysis to quantify the released XMRV and HIV-1 RNA. Results showed that XMRV/HIV-1 co-infected cells released both HIV-1 and XMRV, approximately 2–3×10^9^ RNA genome copies/mL for each virus. Interestingly, both HIV-1 and XMRV release from co-infected cells was three- to four-fold higher compared with virus release from cells infected with HIV-1 or XMRV alone, respectively (Figure 1B).

**Figure 2:**
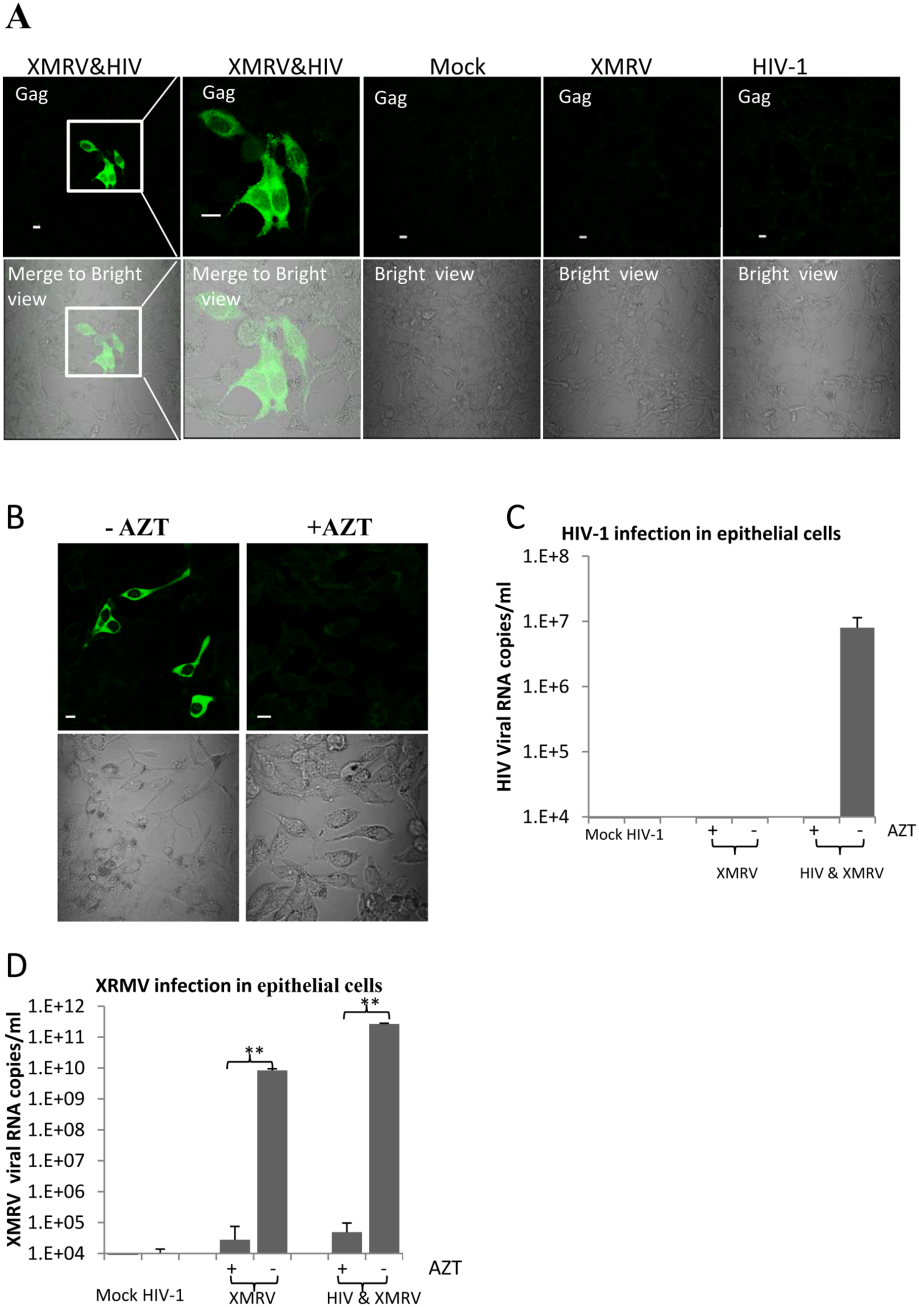
LNcap epithelial cell line is susceptible to infection by HIV-1 co-produced with XMRV. **A.** Visualization of HIV-1 infection in epithelial cells using immunofluorescence staining with anti-HIV Gag monoclonal antibody. LNcap cells were exposed to culture supernatants from CEMX174 cells infected with indicated viruses. Cells were fixed and stained for HIV-1 Gag expression 5 days after exposure to the viral supernatants. Green fluorescence (FITC) indicates HIV-1 Gag expression. Green fluorescence merged with corresponding bright field images are shown in the bottom panels. An enlarged view of cells exposed to HIV-1 co-produced with XMRV is shown in the second panel from the left. **B.** AZT inhibits HIV-1 infection of epithelial cells. LNcap cells and progeny viruses from HIV-1/XMRV co-infected CEMX174 cells were pretreated with AZT or with medium alone. The virus preparations were then added to the cells and the input virus was removed after 24 hrs. The cells were then cultured for an additional four days in medium alone or medium containing AZT as outlined in Materials and Methods. The cells were then fixed and stained for HIV-1 Gag expression. Cells infected in the absence of AZT are shown on the left. The corresponding bright field image is shown in the bottom panels. Bar = 10 µm. Data are representative of 5 independent experiments. **C.** HIV-1 RNA in the supernatants of the same LNcap cells from (**B.**) infected in the presence or absence of AZT was quantified by qRT-PCR. **D.** XMRV RNA in the supernatants of the infected LNcap cells was quantified by qPCR. The input viruses were supernatants from CEMx174 cells infected as indicated. For (C) and (D) the data shown represent the mean ± standard deviation from five independent experiments. **p<0.001, infection in the absence vs presence of AZT.

These data indicate that both HIV-1 and XMRV can replicate and be released efficiently from co-infected T cells. If XMRV glycoprotein is acquired by HIV-1 virions produced in HIV-1/XMRV co-infected T cells, these HIV-1 particles should have acquired XMRV tropism and be able to infect epithelial cells unlike non-pseudotyped HIV-1. To determine if this was true, we used LNcap cells, an epithelial cell line derived from prostate carcinoma that is known to support XMRV replication. We exposed LNcap cells to supernatants from the HIV/XMRV co-infected cells and from cells infected with either HIV-1 alone or XMRV alone. After 5 days, we examined the cells for HIV-1 infection by confocal immunofluorescence microscopy after staining with a MAb against HIV-1 Gag. As showed in Figure 2A (left two panels), LNcap cells were infected by HIV-1 from XMRV/HIV-1 co-infected cells but not by HIV-1 produced alone (Figure 2A, right panel).

To assess whether LNcap cells were productively infected by HIV-1, LNcap cells were infected with progeny viruses from HIV-1/XMRV co-infected T cells in the presence of reverse transcriptase inhibitor azidothymidine (AZT). As shown in Figure 2B, confocal immunofluorescence microscopy showed that HIV-1 infection of LNCap cells could be completely blocked by the antiretroviral drug. This observation was confirmed by real time PCR analysis. HIV-1 viral RNA was only detected in supernatants of cells infected with progeny HIV-1 from XMRV/HIV-1 co-infected T cells (Figure 2C). XMRV replication was confirmed in LNcap cells infected with progeny viruses from both XMRV/HIV-1 co-infected cells and XMRV-infected cells (Figure 2D). AZT treatment not only blocked HIV-1 infection (Figure 2C), but also blocked XMRV infection (Figure 2D), consistent with previous observations [39].

### HIV-1 infection of epithelial cells is inhibited by neutralizing anti-MLV antibody

**Figure 3:**
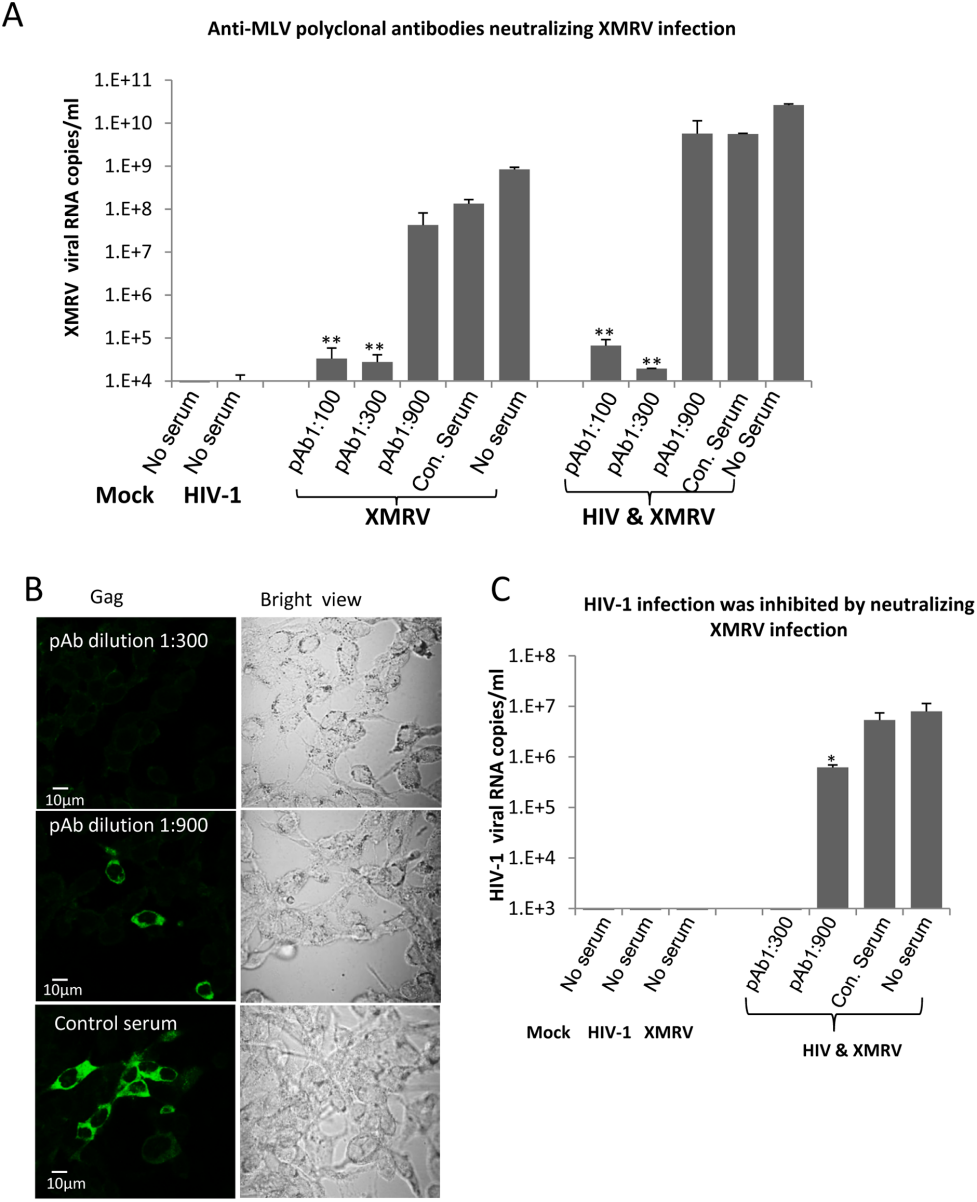
Inhibition of HIV-1 infection of epithelial cells by neutralizing anti-MLV polyclonal antiserum. **A.** Anti-MLV polyclonal antibody neutralizes XMRV infection in a dose-dependent manner. Progeny viruses from CEMX174 cells infected with HIV-1 and/or XMRV were pre-incubated with the indicated dilutions of anti-MLV polyclonal serum at 37°C for 1 hr before addition to LNcap epithelial cells. XMRV released into the supernatants of infected LNcap cells was quantified by qRT-PCR. pAb = Anti-MLV polyclonal antibody; Con. Serum = normal goat serum diluted 1∶100. The input viruses used to infect producer CEMx174 cells are indicated at the bottom. The data shown represent the mean ± standard deviation from three independent experiments. **B.** Immunofluorescence analysis of HIV-1 infection in LNcap cells in the presence or absence of anti-MLV neutralizing antibody. The same LNcap cells infected with progeny viruses from HIV-1/XMRV co-infected cells (shown in A) in the presence of indicated dilutions of anti-MLV neutralizing pAb or control serum were stained for HIV-1 Gag (green). The corresponding bright field image for each panel is shown on the right. Bar = 10 µm. Images are representative of three independent experiments. **C.** HIV-1 viral RNA in supernatants of from the same infected LNcap cells in (B) was quantified by qRT-PCR. The input virus used to infect producer cells and the dilutions of neutralizing or control sera are indicated. The data shown represent the mean ± standard deviation from three independent experiments. *, p<0.05; **, p<0.001, control serum vs anti-MLV pAb.

The data above showing infection of epithelial cells by HIV-1 co-produced with XMRV indicated that co-infection results in HIV-1 bearing the XMRV gp70 glycoprotein. To provide direct evidence that infection of epithelial cells by HIV-1 co-produced with XMRV was mediated through the XMRV glycoprotein, we performed infection assays with a neutralizing polyclonal goat antibody produced against MLV. This polyclonal antiserum has been shown to be highly cross-reactive with XMRV and to neutralize XMRV over a wide dilution range. It does not neutralize HIV-1 [40]. First, we confirmed that XMRV infection is neutralized by the antibody. Progeny viruses from CEMx174 cells co-infected with HIV-1 and XMRV, infected with HIV alone and infected with XMRV alone were incubated with dilutions of heat-inactivated anti-MLV polyclonal sera. After 1 hr at 37°C, the mixtures were added to LNcap epithelial cells. The input virus was removed after 24 hrs and viruses released into the supernatants were analyzed by real-time PCR 5 days post-infection. As shown in Figure 3A, the XMRV viral RNA detected in supernatants was decreased by 4 to 5 logs in the presence of polyclonal antisera against MLV at dilutions of 1∶100 and 1∶300. This was true for control XMRV as well as XMRV co-produced with HIV-1. Infection was not blocked by normal goat serum diluted 1∶100. These data confirmed that neutralizing antibody against MLV effectively neutralized XMRV infection as previously reported [40].

We tested the effect of the anti-MLV antisera on infection of epithelial cells by HIV-1 co-produced with XMRV. The same LNcap cells that were infected with progeny viruses from HIV-1/XMRV co-infected T cells in the presence of the anti-MLV sera were fixed and stained for HIV-1 p24. As shown in Figure 3B, no HIV-1 positive cells were seen when virus was added in the presence of anti-MLV polyclonal sera at a 1∶300 dilution. A few HIV-1 infected cells could be detected at a polyclonal antiserum dilution of 1∶900. HIV-1 positive cells were easily detected when virus was treated with control serum. Consistent with the results from confocal immunofluorescence microscopy, we could not detect HIV-1 viral RNA in culture supernatants from cells exposed to pseudotyped HIV-1 pretreated with anti-MLV sera at a dilution of 1∶300 (Figure 3C). Partial reduction of HIV-1 RNA was observed at an anti-MLV antiserum dilution of 1∶900 (Figure 3C). As expected no HIV-1 viral RNA was detected in supernatants from cells exposed to progeny viruses from cells infected with only HIV-1 or XMRV (Figure 3C). As previously reported, HIV-1 infection of T cells was not blocked by the anti-MLV antiserum (not shown). These data indicate that infection of epithelial cells by HIV-1 co-produced with XMRV is mediated by the XMRV glycoprotein.

### HIV-1 produced from XMRV and HIV-1 co-infected CD4+ T cells infects vaginal and cervical epithelial cell lines

**Figure 4:**
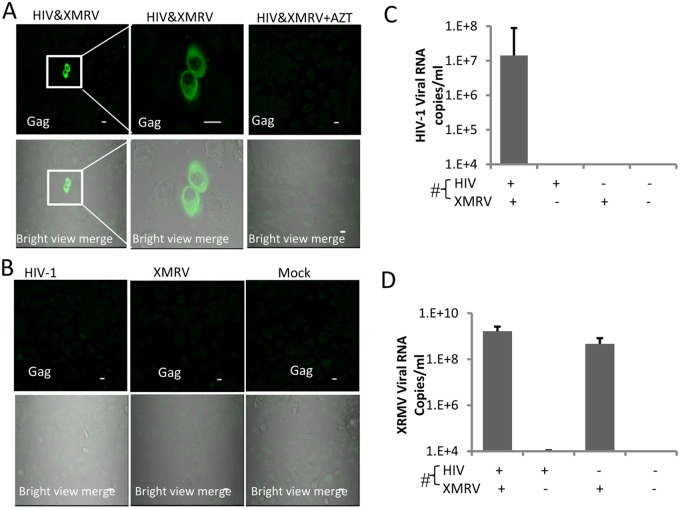
Pseudotyped HIV-1 infects epithelial cells derived from vagina. Immunofluorescence analysis of HIV-1 infection in VK-2. VK-2 cells were infected with progeny virus from primary CD4+ T cells or CEMX174 and were stained for HIV-1 Gag expression 5 days post-infection. The input virus used to infect producer T cells are as indicated. An enlarged view of epithelial cells exposed to progeny virus from HIV-1/XMRV co-infected CEMX174 cells is shown (second panel). HIV-1 Gag expression is indicated by green fluorescence (FITC); green fluorescence merged to the corresponding bright field image is shown in the bottom panels. B. Target epithelial cells were exposed to progeny viruses in the presence or absence of AZT as indicated on the panels. Data shown are representative of six independent experiments. Bar = 10 µm. **C** and **D**, HIV-1 and XMRV viral RNA in the supernatants of the same infected VK-2 cells as in (A) and (B) were quantified by qRT-PCR. The viruses used to infect producer cells are shown on the X axis. The data shown represent the mean ± standard deviation from three independent experiments.

The above results showed that HIV-1 co-produced with XMRV could infect an epithelial cell line. To determine whether female genital epithelial cells could also be directly infected by HIV-1, we employed the vaginal epithelial cell line VK2, derived from normal vaginal tissue. VK2 cells were exposed to progeny viruses from HIV-1/XMRV co-infected T cells for 24 hrs, washed thoroughly, and cultured for an additional 4 days. Cells were then fixed and stained for HIV-1 Gag protein expression. HIV-1 infection was detected in VK2 cells exposed to progeny viruses from HIV-1/XMRV co-infected T cells (Figure 4A). No HIV-1-infected cells were detected in the presence of AZT, confirming active virus replication in VK2 cells (Figure 4A, right panel). We did not detect HIV-1 infection in VK2 cells exposed to progeny viruses from cells infected with HIV-1 alone or XMRV alone or to mock virus (Figure 4B). Q-RT-PCR showed HIV-1 RNA release from VK2 cells exposed to progeny viruses from HIV-1/XMRV co-infected T cells but not virus from control cells (Figure 4C). XMRV viral RNA was detected in supernatants of VK2 cells infected with progeny viruses from both HIV-1/XMRV co-infected T cells and XMRV-infected T cells (Figure 4D).

Both vaginal and cervical epithelia are considered as possible sites for viral entry in sexual transmission of HIV-1 in women. Therefore we tested XMRV-pseudotyped HIV-1 for infection of cervical epithelial cell lines Ect1/E6E7 and Endo1/E6D7. These cell lines were generated from the same normal donor as VK2 cells by *in vitro* HPV E6/E7 immortalization of primary epithelial cells from the ectocervix and endocervix, respectively. These cell lines have been shown to maintain morphologies and phenotypes consistent with their respective tissue origins [35]. HIV-1 Gag specific immunostaining and confocal microscopy showed HIV-1 infection of both Ect1/E6E7 and End1/E6E7 cells when exposed to HIV-1 co-produced with XMRV (Figure S1A, S1B). HIV-1 Gag was not observed in the AZT treated cells and this confirmed active virus replication (Figure S1C, S1D, first panel). No HIV-1 Gag expression was detected in cells exposed to control HIV-1, XMRV or mock virus (Figure S1C, S1D, three panels on the right). These data showed that HIV-1 co-produced with XMRV was able to infect endocervical and ectocervical epithelial cell lines.

**Figure 5:**
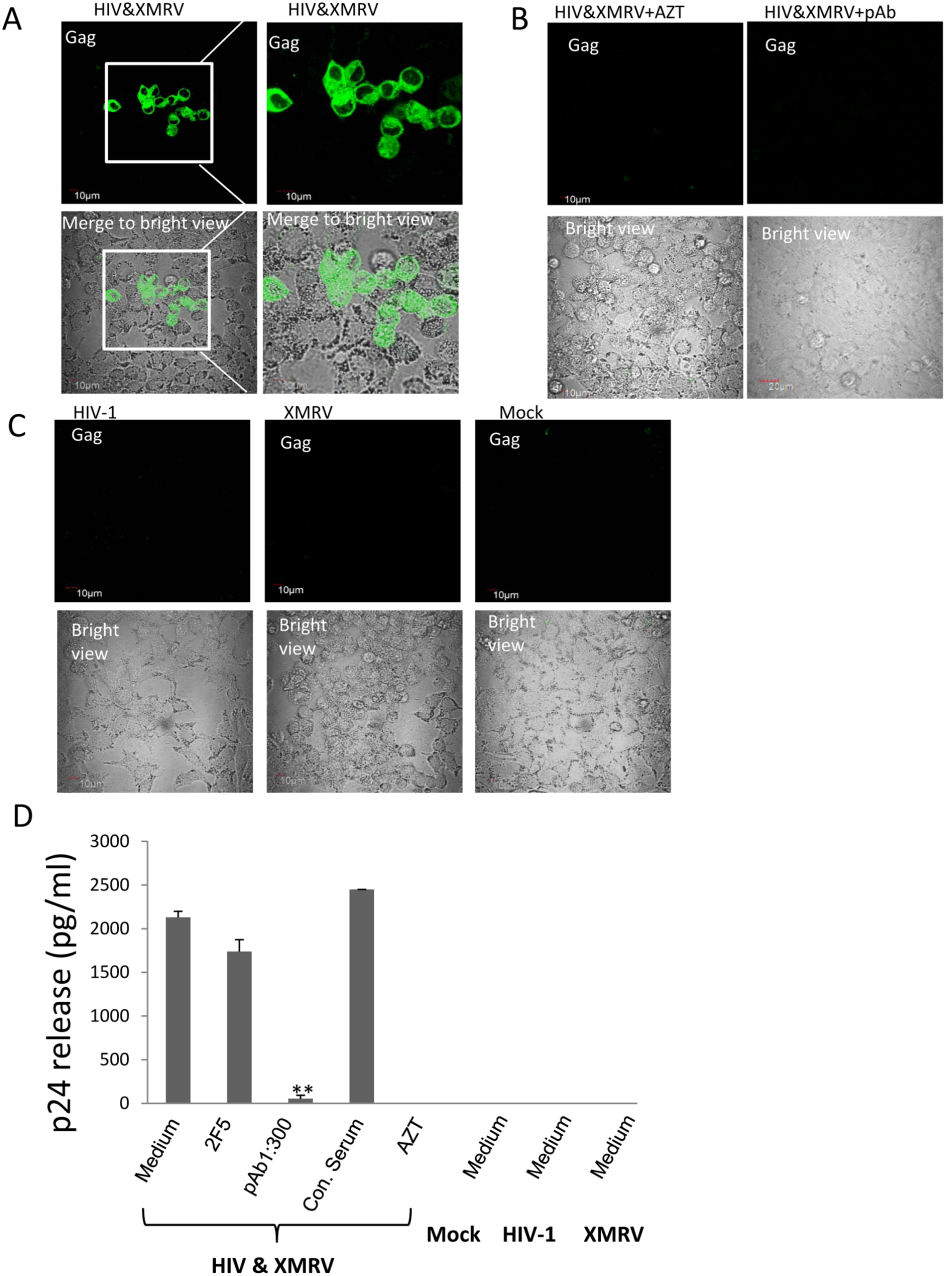
Pseudotyped HIV-1 infects HeLa cells. (**A, C**) Visualization of HIV-1 infection in HeLa cells by immunofluorescence staining with FITC-anti-HIV-1 Gag mAb. HeLa cells were exposed to progeny virus from CEMX174 cells infected with the indicated viruses and immunofluorescence staining was performed with anti-HIV-1 Gag mAb 5 days post-infection. **B**, HeLa cells were exposed to progeny virus from HIV-1/XMRV co-infected CEMX174 cells in the presence of AZT or anti-MLV polyclonal sera (1∶300) and were then stained for HIV-1 Gag expression as above. Anti-Gag fluorescence merged to the corresponding bright field images is shown in the bottom panels. **D**, HIV-1 p24 released into culture supernatants from infected HeLa cells was measured by ELISA. The input virus used to infect producer CEMX174 cells and the treatment of progeny virus used to infect HeLa cells are indicated on the X-axis (pAb 1∶300, anti-MLV polyclonal sera at dilution 1∶300; 2F5, a neutralizing anti-HIV gp120 MAb at concentration of 4 µg/ml; Con. Serum, normal goat serum diluted 1∶300; Medium, medium alone control). **, p<0.001, control serum vs treated samples. The data shown represent the mean ± standard deviation from three independent experiments.

We also tested HIV-1 infection in HeLa cells, a cervical cancer cell line. The cells were exposed to progeny virus from HIV-1 and XMRV co-infected T cells for 24 hrs and after washing away the input virus the cells were cultured for 5 days. The cells were then fixed and stained for HIV-1 Gag expression. As shown in Figure 5A, clusters of HIV-1 positive cells could be easily detected. We used flow cytometry to quantify the percentage of cells that were HIV-1 positive. The data indicated that less than 1% of the cells were HIV-1 infected (Figure S2A), consistent with a low frequency of pseudotyped HIV-1 particles. HIV-1 infection in HeLa cells was also assessed by HIV-1 p24 ELISA analysis of cell supernatants. HIV-1 was detected in supernatants from HeLa cells exposed to progeny virus from HIV-1/XMRV co-infected T cells (Figure 5D). In contrast, HIV-1 infection was not observed in HeLa cells exposed to virus released from T cells infected with XMRV or HIV-1 alone (Figure 5C, D). The infection of HeLa cells by HIV-1 was blocked by AZT and neutralizing anti-MLV sera (Figure 5B, D). Control non-immune serum and a well-characterized neutralizing anti-HIV-1 gp120 antibody 2F5 (4 µg/ml) had no effect on infection of HeLa cells by pseudotyped HIV-1 (Figure 5D). To confirm 2F5 neutralization of HIV-1, we performed a parallel experiment in TZM-bl cells, which are HeLa cells expressing endogenous CXCR4 that have been stably transfected with CD4 and CCR5. These cells efficiently support HIV-1 infection and replication [41]. HIV-1 produced in CEMX174 cells was incubated for 1 hr with serial dilutions of 2F5 MAb before adding the virus to the cells. Infection of TZM-bl cells by HIV-1 was blocked by 2F5 at concentrations of 2 ug/ml or higher (Figure S2B). These results confirm data shown above indicating that HIV-1 infection in epithelial cells is mediated by XMRV glycoprotein acquired by HIV-1 during co-infection with XMRV.

### XMRV pseudotyped HIV-1 infects primary female lower genital epithelial cells

The human female lower genital tract is lined with two types of mucosal epithelial cells, columnar and squamous. Columnar epithelial cells form a single layer lining the surface of the endocervix and stratified squamous epithelial cells form the mucosal lining of the vagina and ectocervix. These epithelial cells are the first cells that HIV-1 encounters in the female genital tract and they provide a natural barrier to prevent HIV-1 transmission. This barrier would be potentially ineffective if HIV-1 acquires the glycoprotein of a virus with tropism for epithelial cells. We thus sought to determine if HIV-1 co-produced with XMRV could infect primary epithelial cells from the female lower genital tract.

To establish primary epithelial cell cultures, fresh human endocervical, ectocervical and vaginal tissue were collected, cut into 1×1 cm pieces and placed in culture in epithelial cell growth medium. Epithelial cells began migrating into the culture vessels after 2 days and migration peaked after 3 to 5 days (Figure S3A). Monolayers of primary cells were well-established by day 7. The endocervical columnar epithelial cells displayed a flattened cobble-stone morphology as previously noted [35], [42] (Figure S3B). Immunostaining and confocal microscopy showed that endocervix-derived epithelial cells expressed high levels of cytokeratins CK8 and CK19 (Figure S3D), but low levels of involucrin. Vaginal and ectocervical squamous epithelial cells displayed the expected flattened, stretched morphology (Figure S3C). Immunostaining and confocal microscopy indicated that these cells expressed high levels of cytokeratin CK19. In contrast to the primary endocervical cells, the squamous vaginal and ectocervical epithelial cells expressed low levels of CK8 and high levels of involucrin (Figure S3E). These data confirmed that the epithelial cells isolated from normal tissues had marker phenotypes reflecting their tissue of origin and our phenotyping data are consistent with those in a previous report [35]. While both vaginal and cervical primary epithelial cells expressed the HIV-1 co-receptor CXCR4, they did not express hematopoietic markers including the HIV-1 receptor CD4 and co-receptor CCR5 (Figure S3F, S3G).

**Figure 6:**
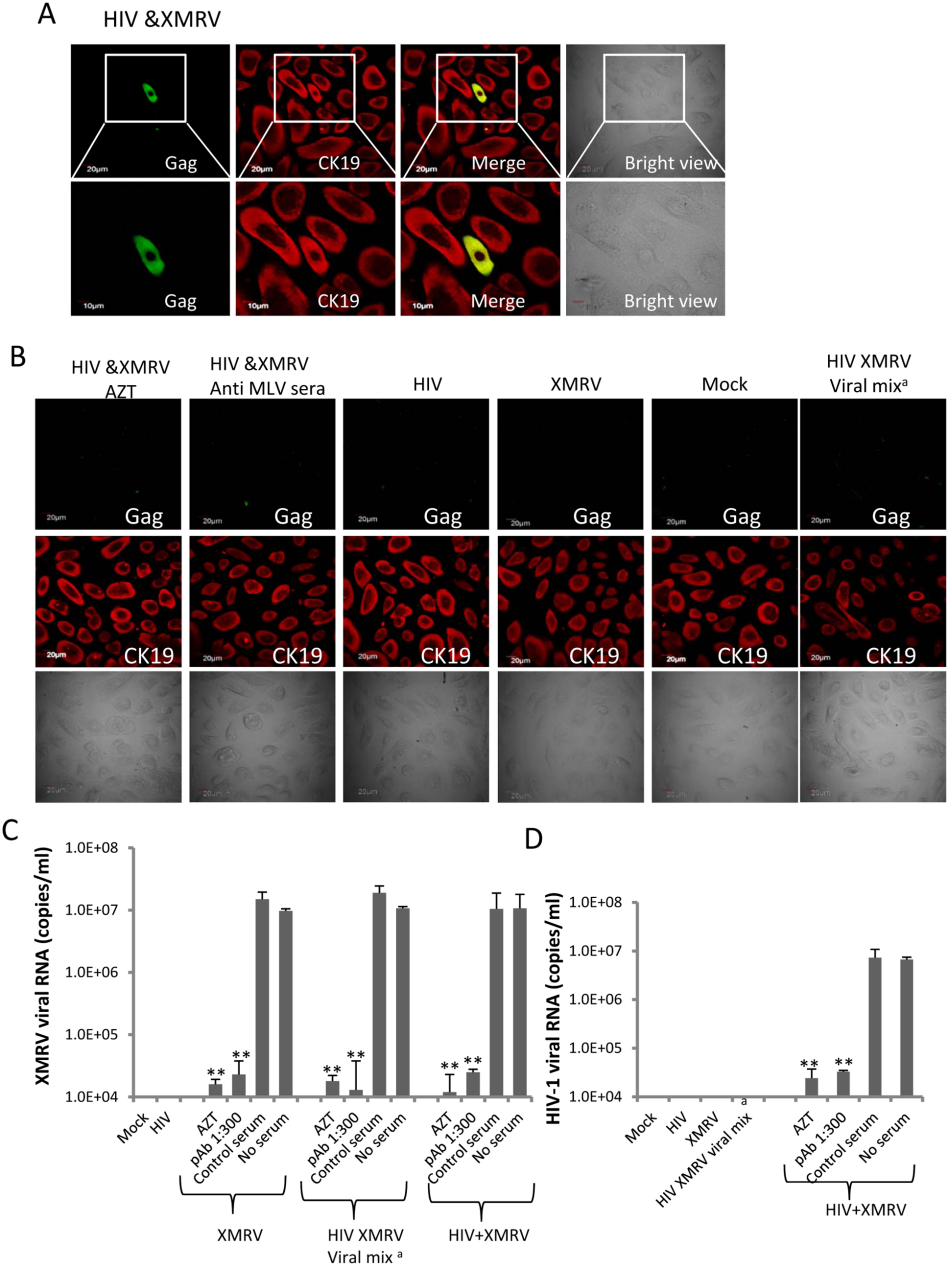
Pseudotyped HIV-1 infects primary endocervical epithelial cells. (**A, B**) Visualization of HIV-1 infection in primary endocervical epithelial cells by dual immunofluorescence staining with FITC-anti-HIV-1 Gag and anti-CK19 MAbs. Primary endocervical epithelial cells were exposed to progeny viruses from infected CEMX174 cells and immunofluorescence staining was performed 5 days post-infection. Epithelial cells exposed to virus in presence of AZT or anti-MLV polyclonal sera diluted 1∶300 are shown as indicated (**B**, left two panels). HIV-1 Gag fluorescence is shown as green and CK19 as red. The corresponding bright field images are shown on the right (**A**) or at the bottom (**B**). HIV-1 Gag staining merged with CK19 staining and enlarged views of HIV-1 infected cells are shown in (A). **C and D:** XMRV RNA (**C**) and HIV-1 RNA (**D**) in supernatants from the same infected primary endocervical epithelial cells from (**A**) and (B) above were quantified by qRT-PCR. The input virus used to infect producer CEMX174 cells and the treatments are indicated on the X-axis of the graphs (pAb 1∶300 = anti-MLV polyclonal sera at dilution 1∶300; Control serum = normal goat serum diluted 1∶300; No serum = culture medium control). **, p<0.001, control serum vs indicated treatments. The data shown represent the mean ± standard deviation from three independent experiments. **^a^**, HIV-1 and XMRV from CEMX174 cells infected with each virus alone were quantified and mixed at a ratio equal to the ratio of the two viruses in the progeny virus from HIV-1 and XMRV co-infected CEMX174 cells. The virus mixture was then inoculated onto primary endocervical epithelial cells and immunostaining and qPCR was performed exactly as described for progeny virus from co-infected CEMx174 cells.

The primary cells were exposed to progeny virus from CEMx174 cells infected with HIV-1 alone (HIV), XMRV alone (XMRV), or both HIV-1 and XMRV (HIV/XMRV). The input of HIV-1 (RNA genomes) was kept constant and supernatant from uninfected CEMx174 cells was used as a control (mock). We also set up another control in which we pre-mixed equivalent amounts of wild type HIV-1 and XMRV (both from singly infected cell cultures) as found in progeny virus from co-infected cells and exposed epithelial cells to the virus mixture. The progeny viruses and virus mixtures were left on the cell monolayers for 24 hrs and then removed by extensive washing. Fresh medium was then added and the cells were cultured for an additional 4 days. Viral infection in epithelial cells was detected by confocal immunofluorescence microscopy after MAb staining with anti-HIV-1 Gag. As shown in Figure 6A, we observed HIV-1 infection of primary endocervical epithelial cells that were exposed to HIV-1 co-produced with XMRV (green staining). Expression of the CK19 marker (red) confirmed that the infected cells were epithelial cells. HIV-1 infection was inhibited by AZT or by pre-treatment of virus with neutralizing anti-MLV polyclonal sera (Figure 6B, left two panels). These data showed that productive HIV-1 infection of endocervical cells occurred that required XMRV gp70-mediated entry. No HIV-1 infection was detected in cells exposed to virus from CEMX174 cells infected with HIV-1 alone or XMRV alone (Figure 6B). Notably, mixtures of equivalent amounts of wild type HIV-1 and XMRV did not lead to HIV-1 infection of the epithelial cells. This indicated that XMRV infection of epithelial cells did not lead to phenotypic or other changes that rendered the cells permissive for HIV-1 infection (Figure 6B, right panel).

**Figure 7:**
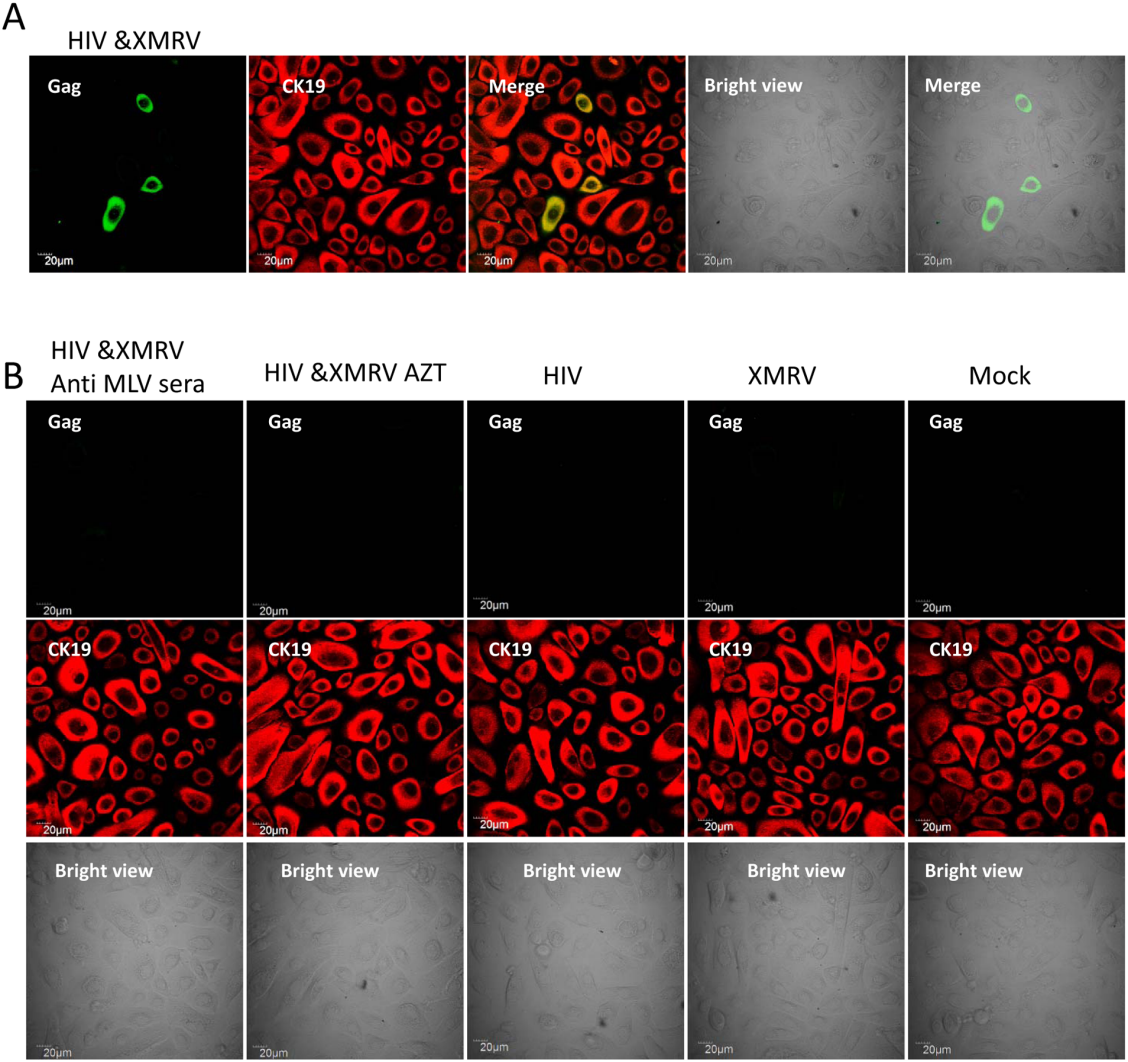
Pseudotyped HIV-1 infects primary vaginal squamous epithelial cells. (**A, B**) Dual immunofluorescence staining with FITC-anti-HIV-1 Gag and anti-CK19 MAbs was performed on primary vaginal squamous epithelial cells which were exposed to progeny virus from infected CEMX174 cells. HIV-1 Gag is shown as green and CK19 is shown as red. Corresponding bright field images are also shown as indicated. The input viruses used to infect producer CEMX174 cells is indicated on the panels. Epithelial cells exposed to progeny virus in the presence of AZT or anti-MLV polyclonal sera diluted 1∶300 are shown in the left two columns in **B**.

To further confirm the above observations, we examined virus release in supernatants from infected endocervical epithelial cells by quantitative PCR. Equivalent amounts of XMRV viral RNA was released from endocervical cells exposed to XMRV, XMRV and HIV-1 mixtures and progeny virus from HIV-1/XMRV co-infected T cells respectively (Figure 6C). In contrast to XMRV, HIV-1 viral RNA was only detected in supernatants from endocervical epithelial cells that were exposed to progeny virus from HIV-1 and XMRV co-infected cells. HIV-1 RNA was not detected in supernatants from cells exposed to control HIV-1 or to HIV-1 and XMRV mixtures (Figure 6D). XMRV infection was inhibited by AZT and by anti-MLV polyclonal serum (Figure 6C). PCR data confirmed that AZT treatment and anti-MLV polyclonal serum inhibited HIV-1 infection in epithelial cells exposed to progeny virus from HIV-1 and XMRV co-infected cells (Figure 6D). We also determined whether primary vaginal epithelial cells could be infected by naturally pseudotyped HIV-1. The primary vaginal epithelial cells were exposed to progeny virus from HIV-1/XMRV co-infected CEMX174 cells, from CEMX174 cells infected with either virus alone, or from mock infected control cells under the same conditions as described above. At 5 days post-infection epithelial cells were subjected to immunostaining for expression of HIV-1 Gag and the epithelial marker CK19. The data revealed that primary vaginal epithelial cells were infected by HIV-1 after exposure to progeny virus from HIV-1/XMRV co-infected T cells (Figure 7A). This infection was blocked by AZT and by neutralizing anti-MLV polyclonal serum (Figure 7B, left two panels). No HIV-1 infection was detected in the epithelial cells exposed to control HIV-1, XMRV or mock virus (Figure 7B). Data obtained in primary ectocervical epithelial cells was similar to that obtained in primary vaginal epithelial cells (data not shown).

**Figure 8:**
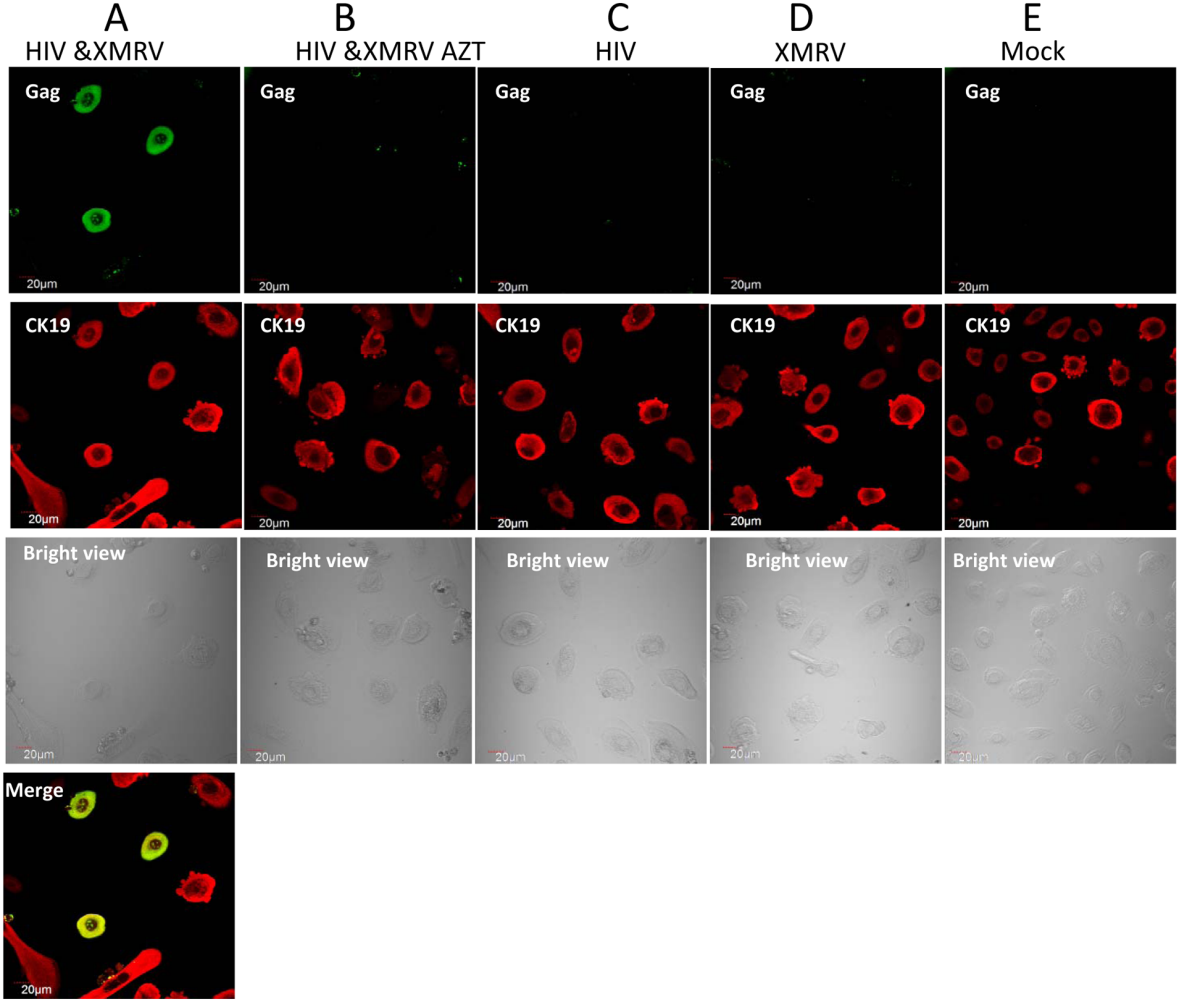
Pseudotyped HIV-1 infection of primary endocervical cells via cell-associated virus. Primary endocervical epithelial cells were co-cultured for two days with an equal number of mitomycin C-treated CEMX174 cells infected with HIV-1 alone, XMRV alone or co-infected with both viruses. After washing to remove non-adherent cells, dual immunofluorescence staining with FITC-anti-Gag and anti-CK19 MAbs was performed on the adherent primary endocervical epithelial cells on day 5. Merged images confirmed that HIV-1 infected cells were epithelial cells. Primary endocervical epithelial cells were co-cultured with (A) HIV-1/XMRV co-infected CEMX174; (B) same as (A) in presence of AZT; (C) HIV-1 infected CEMx174; (D) XMRV infected CEMx174; (E) mock infected CEMx174.

Published data indicates that sexual transmission of HIV-1 may be mediated by cell-associated virus [43]–[45]. Therefore we determined whether HIV-1 infection of primary epithelial cells could be mediated by T cells co-infected with HIV-1 and XMRV. Infected CEMX174 cells were pre-treated with mitomycin C and the cells were then co-cultured with primary epithelial cells for 2 days. The non-adherent CEMx174 cells were removed by washing and HIV-1 Infection in the epithelial cells was assessed by confocal immunomicroscopy. The data revealed HIV-1 Gag protein expression in primary endocervical epithelial cells that were co-cultured with HIV-1/XMRV co-infected CEMX174 cells (Figure 8A). The infection was blocked by AZT (Figure 8B). No HIV-1 infection was detected in epithelial cells co-cultured with CEMx174 cells infected with HIV-1 (C), XMRV (D) or mock infected (E).

Previous reports suggest that sexual transmission of HIV-1 may primarily involve strains that utilize CCR5 as co-receptor (R5 tropic) [46]–[47]. We repeated the above experiments with the R5-tropic HIV-1 strain HIV-1BaL. The data showed that HIV-1BaL co-produced with XMRV infected primary vaginal epithelial cells (Figure S4). Thus our data indicates that both X4 and R5 HIV-1 strains can infect primary genital epithelial cells after natural pseudotyping.

## Discussion

Phenotypic intermixing of viral components or pseudotyping is a well-known phenomenon first described in the 1970’s [48], [49]. In the1990s, several studies indicated that when produced in cells infected with a second virus, including HSV [57], VSV [58], HTLV [59] and MLV [60], HIV-1 appeared to have an expanded host range, suggesting that pseudotyped virions had formed. Subsequent studies demonstrated that pseudotyped HIV-1 particles were produced when heterologous virus glycoproteins were expressed in cells transfected with cloned HIV-1 DNA [50], [61]–[63]. Pseudotyping is now routinely employed to target viral vectors for gene delivery to specific cells and has been applied to a wide variety of enveloped viruses including retroviruses, herpesviruses and rhabdoviruses [50]–[54]. Our previous work demonstrated that HIV-1 can acquire host proteins expressed in the membrane of infected cells [55], [56]. Cholesterol-rich membrane microdomains (lipid rafts) are the sites for HIV-1 assembly and proteins present in lipid rafts are selectively incorporated by the virus [27], [28], [30], [31], [52]. Glycoproteins from other viruses that traffic to lipid rafts are therefore predicted to be incorporated by HIV-1 and published data confirm this prediction [51]. A further prediction of these observations was that HIV-1 could be pseudotyped when it co-infects cells with certain other viruses. We call this process “natural pseudotyping” to distinguish it from *in vitro* pseudotyping achieved through molecular genetics techniques as described above. In the current study we have used XMRV as a model second virus to demonstrate that natural pseudotyping of HIV-1 occurs resulting in expansion of HIV-1 tropism to include primary genital epithelial cells.

During the past 30 years very few studies have examined the significance or potential implications of natural pseudotyping in viral transmission or pathogenesis. One potential reason why natural pseudotyping studies have been largely discontinued is the technical challenge of obtaining biochemical and ultrastructural evidence to support phenotypic mixing of viral particles. Indeed, several investigators have reported that they could not detect pseudotyped HIV-1 particles after co-infecting cells with HIV-1 and HSV or other viruses [64]–[67]. In some of these studies [64], virus capture or pull-down assays were often used to detect pseudotyped HIV-1. In such assays an antibody against the heterologous glycoprotein is used to precipitate pseudotyped HIV-1 and then HIV-1 Gag protein or RNA is measured to confirm the presence of HIV-1 particles. In other studies PCR was used to measure HIV-1 replication in CD4-negative cells exposed to progeny HIV-1 from co-infected cells. This more sensitive assay also failed to detect HIV-1 infection in CD4-negative cells [65]–[67]. If the frequency of pseudotyped HIV-1 particles is extremely low, even PCR assays may not be sensitive enough to detect infection by the pseudotyped virus. We used primary T cells and CEMx174 cells as producer cells and found that the two co-infecting viruses (HIV-1 and XMRV) replicated very well in these cells increasing the likelihood that pseudotyping would occur. In addition, we employed a biological (infection) assay to detect the presence of pseudotyped HIV-1 since even a low frequency of infection by pseudotyped viruses would result in amplification of viral proteins and RNA. Our data showed that the frequency of naturally pseudotyped HIV-1 was indeed low but sufficient to result in measurable infection. To rule out endocytic uptake of HIV-1 by these cells [68], [69], we confirmed active replication with the RT inhibitor AZT. Furthermore, neutralization by an MLV-neutralizing antiserum showed that HIV-1 infection of epithelial cells was mediated by the XMRV glycoprotein.

The risk of HIV-1 transmission during sexual intercourse has been reported to be as low as 0.1% [4], [5]. Genital epithelial cells do not express receptors for HIV-1 and represent a mechanical barrier to virus entry. Our results confirmed that primary female genital epithelial cells are not susceptible to infection by WT HIV-1. However, when co-produced with a second virus with tropism for epithelial cells, progeny HIV-1 were able to infect primary vaginal and endocervical epithelial cells. These results were highly reproducible and indicate that natural pseudotyping of HIV-1 has the potential to drastically increase the risk of sexual transmission by rendering the virus capable of infecting genital epithelial cells that normally serve as barriers to virus entry. A number of factors can substantially increase the risk of sexual transmission of HIV-1, including mucosal ulceration, inflammation, and pre-existing sexually transmitted infections. The increase in risk of infection associated with these conditions maybe stochastic; i.e., increased numbers of HIV-1-susceptible cells in the genital tract resulting in increased likelihood that virus particles will find a target cell to infect [70]–[72]. In the case of a naturally pseudotyped HIV-1 particle able to infect genital epithelial cells directly, particularly endocervical cells, increased risk of transmission would not necessarily depend on any of the above factors. The risk of infection for each exposure to pseudotyped HIV-1 may be quite high. Once HIV-1 has infected genital epithelial cells it would revert back to its WT tropism since it acquires the heterologous viral envelope protein but not its gene during natural pseudotyping. The HIV-1-infected epithelial cells could spread the virus to intraepithelial T cells, macrophages or dendritic cells that are normally present in genital tissues and this would then result in systemic infection after these cells migrate to lymphoid tissues [73]–[75]. Interestingly, others have shown that epithelial cells infected with HIV-1 and other pathogenic viruses release proinflammatory cytokines [76]–[78]. Thus it is possible that after infection of genital epithelial cells by pseudotyped HIV-1 and or the second epithelial-tropic virus from the transmitting host, release of such factors would result in influx of HIV-1-susceptible T cells and macrophages thereby increasing the likelihood of hematopoietic spread of the virus.

We used the gammaretrovirus XMRV as the model virus for proving that natural pseudotyping could result in infection of primary genital cells by HIV-1. At the time our work began XMRV was thought to be a potential human pathogen but subsequent studies disproved this notion [25] and it is unlikely that XMRV is relevant to HIV-1 biology in humans [79]. However, the majority of individuals infected with HIV-1 are co-infected by one of several other pathogenic viruses including HTLV I/II [18], [80], HBV, HCV [19], [81], GBV-C [21], HCMV [82], HHV-6/7/8, EBV [83], and HSV-1/2 [22], [84]. As noted above, *in vitro* studies have shown that HIV-1 can be pseudotyped with envelope glycoproteins from several of these viruses [50], [57]–[59]. Also, recent publications show that human endogenous retroviruses (HERV) can be activated after HIV-1 infection [85], [86] and it has been demonstrated that HIV-1 can be pseudotyped by a HERV glycoprotein [87]. It is noteworthy that many of these pathogenic viruses, including HCMV and HTLV, have a tropism that overlaps with that of HIV-1 and they are able to infect T cells and or macrophages [88]–[91]. Thus it is possible that natural pseudotyping of HIV-1 occurs *in vivo*, especially in regions with high prevalence of other human pathogenic viruses. In southern Africa and other regions of the world with high HIV-1 transmission rates, prevalence of viruses such as HTLV-1/2 and CMV is also high [18], [80], [82], [92]–[94]. Natural pseudotyping of HIV-1 with glycoproteins from these viruses may be one of several factors responsible for high HIV-1 transmission rates. Natural pseudotyping may also be a factor in transmission of HIV-1 by so-called “superspreaders” since co-infection with other sexually transmitted viruses and other pathogens has been shown in HIV-1 superspreaders [8], [70], [71]. Interestingly, a past study reported that co-infection with HTLV can affect disease progression in HIV-1 infected patients [80]. The expanded tropism of HIV-1 as a consequence of pseudotyping and infection of non-hematopoietic cells could possibly explain, at least in part, changes in disease progression in HIV-1/HTLV co-infected individuals. The ability of HIV-1 to change its tropism by acquiring glycoproteins from other viruses or endogenous retroviruses could also explain published reports of infection of non-CD4+ cells *in vivo*, including epithelial cells and hepatocytes [95]–[98]. Interestingly, there have been reports that efforts to reduce the prevalence of HSV also resulted in lower infection rates of HIV-1 [99], [100].

This work has potentially important implications for sexual transmission of HIV-1. The mechanisms involved in sexual transmission of HIV-1 remain unclear. There is no evidence of direct infection of genital epithelial cells by HIV-1 as a mode of viral transmission. There is also no evidence proving otherwise and an increasing number of reports indicate that epithelial cells from genital and other tissues can be infected by the virus [95]–[98]. As noted above, very low frequency infection of epithelial cells by pseudotyped HIV-1 could result in rapid spread to intraepithelial hematopoietic cells. So it is possible that an apparent transmission event involving CD4+ cells actually resulted from an initial infection of epithelial cells. We suggest that natural pseudotyping could play an important role in HIV-1 transmission in some settings and warrants further investigation.

## Supporting Information

Figure S1 **Pseudotpyed HIV-1 infects epithelial cells derived from the cervix.** Immunofluorescence analysis of HIV-1 infection in Ect1/E6E7 (A, C) and End1/E6E7 (B, D) cells. Ect1/E6E7 and End1/E6E7 cells were infected with progeny virus from primary CD4+ T cells or CEMX174 and were stained for HIV-1 Gag expression 5 days post-infection. The input virus used to infect producer T cells are as indicated; target epithelial cells exposed to progeny viruses in presence of AZT are indicated on the panels (shown in (C) and (D)). An enlarged view of epithelial cells exposed to progeny virus from HIV-1/XMRV co-infected CEMX174 cells is shown (second panel of A, B). HIV-1 Gag expression is indicated by green fluorescence (FITC); green fluorescence merged to the corresponding bright field image is shown in the bottom panels. Data shown are representative of six independent experiments. Bar = 10 µm.
(TIF)

Figure S2 **HIV-1 infection of HeLa cells and neutralizing activity of 2F5 MAb.** (A) Quantifying HIV-1 infection in HeLa cells. HeLa cells were exposed to progeny virus from CEMx174 cells infected with HIV alone (HIV-1), XMRV alone (XMRV) or co-infected with both (HIV/XMRV). Supernatant from uninfected CEMx174 cells (mock) was used as a control. Immunofluorescence staining and flow cytometry analysis were then performed with FITC-anti-HIV-1 Gag MAb. HeLa cells were also exposed to infected by the progeny virus from HIV/XMRV co-infected cells in presence of AZT (second panel). (B) The neutralizing activity of 2F5 antibody against HIV-1 was confirmed by exposing TZM-bl cells to HIV-1 in the presence of dilutions of the antibody. Infection was assessed after 2 days by measuring luciferase activity as described in Materials and Methods.
(TIF)

Figure S3 **Isolation and characterization of primary cervical and vaginal epithelial cells.** (**A**) Representative image to show epithelial cells migrating from tissue explants after 5 days of culture. Endocervix (**B**) and vagina (**C**) derived epithelial cells formed monolayers after 7 days of culture. **D, E, F, G:** The epithelial cells from endocervix (**D, F**) or vagina and ectocervix (**E, G respectively**) were subjected to immunofluorescence staining for the indicated protein as described in Materials and Methods.
(TIF)

Figure S4 **Visualization of R5 strain HIV-1Bal infection of primary endocervical epithelial cells.** Dual immunofluorescence staining with FITC-anti-HIV-1 Gag and anti-CK19 Mabs was performed in primary endocervical epithelial cells that were exposed to progeny virus from infected CEMx174 cells. The input viruses used to infect CEMx174 cells are indicated (HIV = HIV alone; XMRV = XMRV alone; HIV/XMRV = co-infected with both). Epithelial cells exposed to progeny virus in presence of AZT or anti-MLV polyclonal sera diluted 1∶300 are shown as indicated (**B**, left two columns). HIV-1 Gag is shown as green and CK19 as red. Green fluorescence merged to the corresponding bright field is shown in (**A**).
(TIF)
