# Supplementary figures and images for: Infection of Female Primary Lower Genital Tract Epithelial Cells after Natural Pseudotyping of HIV-1: Possible Implications for Sexual Transmission of HIV-1
Source: PLoS One. 2014 Jul 10;9(7):e101367. doi: 10.1371/journal.pone.0101367 (PMC4092063; doi:10.1371/journal.pone.0101367)

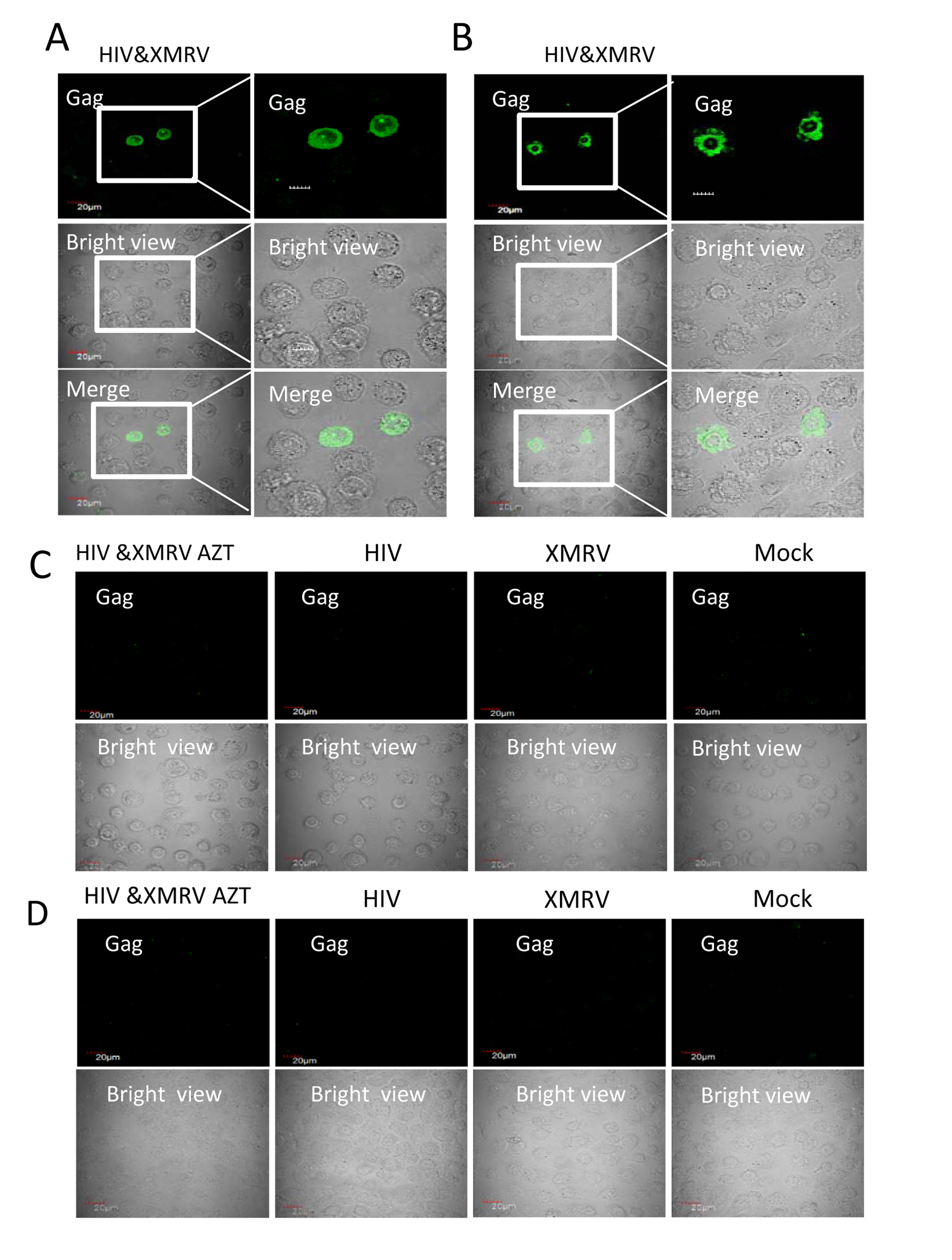

Supplement: Figure S1 — Pseudotpyed HIV-1 infects epithelial cells derived from the cervix. Immunofluorescence analysis of HIV-1 infection in Ect1/E6E7 (A, C) and End1/E6E7 (B, D) cells. Ect1/E6E7 and End1/E6E7 cells were infected with progeny virus from primary CD4+ T cells or CEMX174 and were stained for HIV-1 Gag expression 5 days post-infection. The input virus used to infect producer T cells are as indicated; target epithelial cells exposed to progeny viruses in presence of AZT are indicated on the panels (shown in (C) and (D)). An enlarged view of epithelial cells exposed to progeny virus from HIV-1/XMRV co-infected CEMX174 cells is shown (second panel of A, B). HIV-1 Gag expression is indicated by green fluorescence (FITC); green fluorescence merged to the corresponding bright field image is shown in the bottom panels. Data shown are representative of six independent experiments. Bar = 10 µm. (TIF) [file pone.0101367.s001.tif]

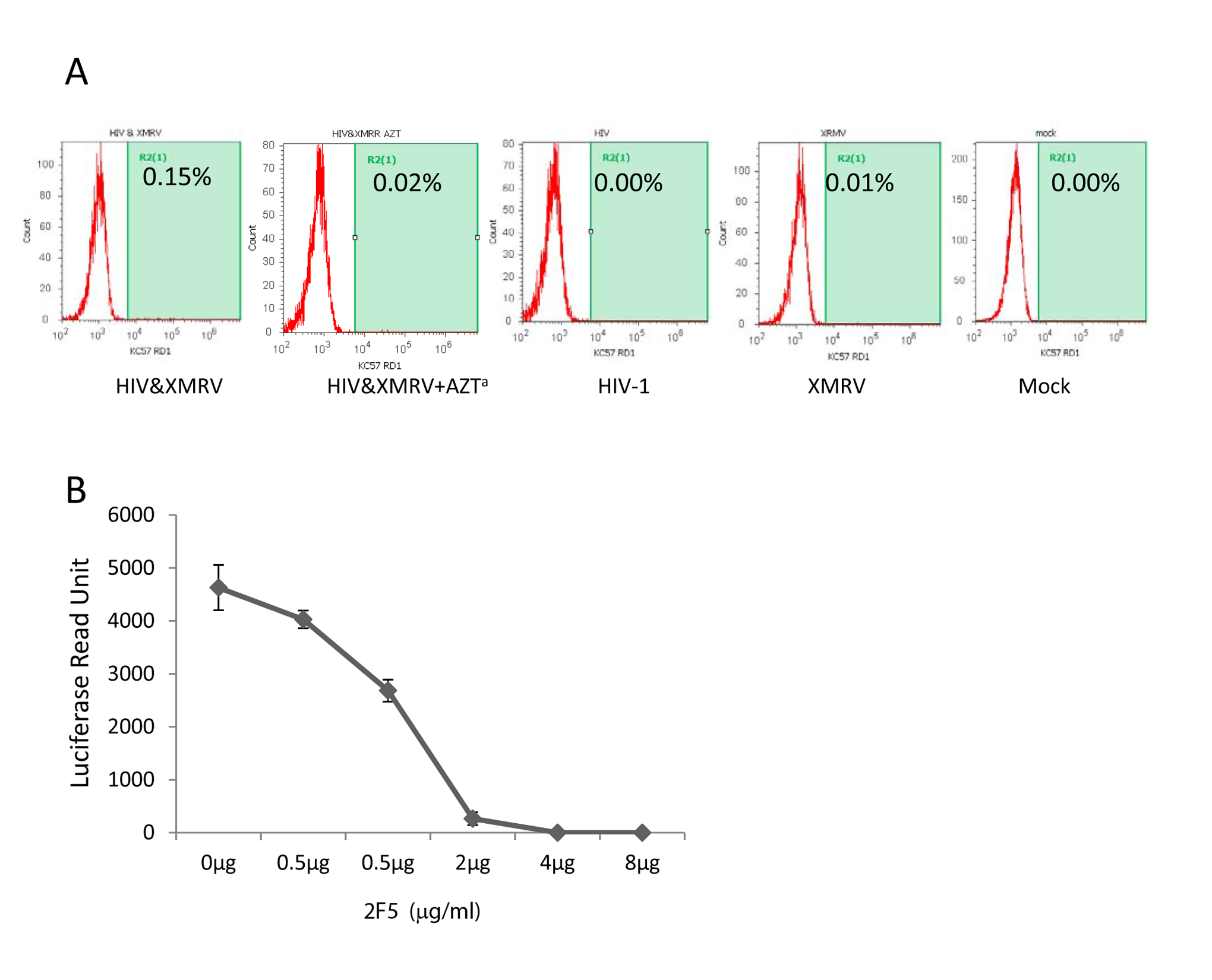

Supplement: Figure S2 — HIV-1 infection of HeLa cells and neutralizing activity of 2F5 MAb. (A) Quantifying HIV-1 infection in HeLa cells. HeLa cells were exposed to progeny virus from CEMx174 cells infected with HIV alone (HIV-1), XMRV alone (XMRV) or co-infected with both (HIV/XMRV). Supernatant from uninfected CEMx174 cells (mock) was used as a control. Immunofluorescence staining and flow cytometry analysis were then performed with FITC-anti-HIV-1 Gag MAb. HeLa cells were also exposed to infected by the progeny virus from HIV/XMRV co-infected cells in presence of AZT (second panel). (B) The neutralizing activity of 2F5 antibody against HIV-1 was confirmed by exposing TZM-bl cells to HIV-1 in the presence of dilutions of the antibody. Infection was assessed after 2 days by measuring luciferase activity as described in Materials and Methods. (TIF) [file pone.0101367.s002.tif]

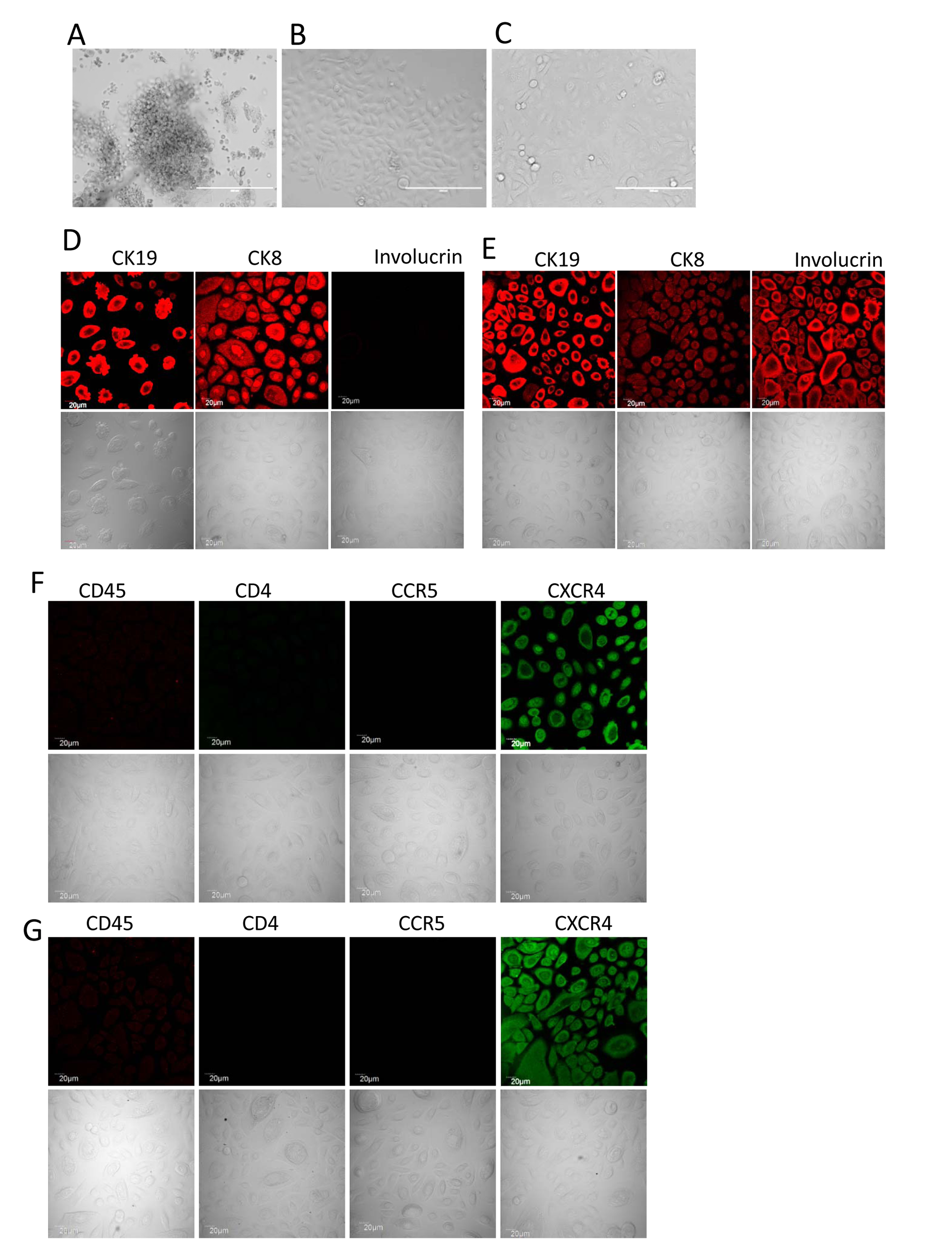

Supplement: Figure S3 — Isolation and characterization of primary cervical and vaginal epithelial cells. (A) Representative image to show epithelial cells migrating from tissue explants after 5 days of culture. Endocervix (B) and vagina (C) derived epithelial cells formed monolayers after 7 days of culture. D, E, F, G: The epithelial cells from endocervix (D, F) or vagina and ectocervix (E, G respectively) were subjected to immunofluorescence staining for the indicated protein as described in Materials and Methods. (TIF) [file pone.0101367.s003.tif]

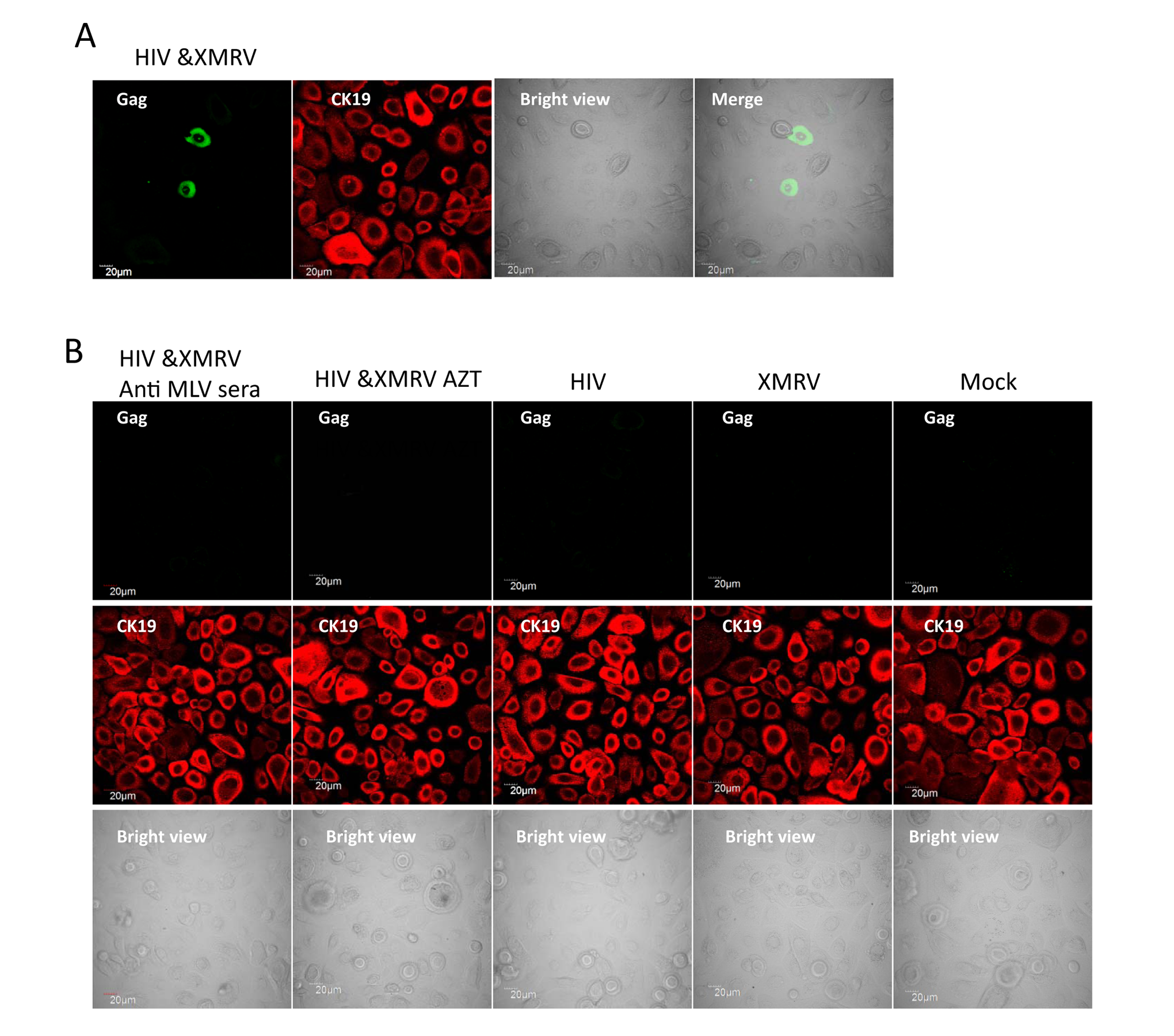

Supplement: Figure S4 — Visualization of R5 strain HIV-1Bal infection of primary endocervical epithelial cells. Dual immunofluorescence staining with FITC-anti-HIV-1 Gag and anti-CK19 Mabs was performed in primary endocervical epithelial cells that were exposed to progeny virus from infected CEMx174 cells. The input viruses used to infect CEMx174 cells are indicated (HIV = HIV alone; XMRV = XMRV alone; HIV/XMRV = co-infected with both). Epithelial cells exposed to progeny virus in presence of AZT or anti-MLV polyclonal sera diluted 1∶300 are shown as indicated (B, left two columns). HIV-1 Gag is shown as green and CK19 as red. Green fluorescence merged to the corresponding bright field is shown in (A). (TIF) [file pone.0101367.s004.tif]
